# Chemokine-mediated inflammation in the degenerating retina is coordinated by Müller cells, activated microglia, and retinal pigment epithelium

**DOI:** 10.1186/s12974-014-0224-1

**Published:** 2015-01-17

**Authors:** Matt Rutar, Riccardo Natoli, RX Chia, Krisztina Valter, Jan M Provis

**Affiliations:** John Curtin School of Medical Research, The Australian National University, Building 131, Garran Road, Canberra, ACT 2601 Australia; ANU Medical School, The Australian National University, 54 Mills Road, Canberra, ACT 2601 Australia

## Abstract

**Background:**

Monocyte infiltration is involved in the pathogenesis of many retinal degenerative conditions. This process traditionally depends on local expression of chemokines, though the roles of many of these in the degenerating retina are unclear. Here, we investigate expression and *in situ* localization of the broad chemokine response in a light-induced model of retinal degeneration.

**Methods:**

Sprague–Dawley (SD) rats were exposed to 1,000 lux light damage (LD) for up to 24 hrs. At time points during (1 to 24 hrs) and following (3 and 7 days) exposure, animals were euthanized and retinas processed. Microarray analysis assessed differential expression of chemokines. Some genes were further investigated using polymerase chain reaction (PCR) and *in situ* hybridization and contrasted with photoreceptor apoptosis using terminal deoxynucleotidyl transferase dUTP nick-end labeling (TUNEL). Recruitment of retinal CD45^*+*^ leukocytes was determined via fluorescence activated cell sorting (FACS), and expression of chemokine receptors determined using PCR.

**Results:**

Exposure to 24 hrs of LD resulted in differential expression of chemokines including Ccl3, Ccl4, Ccl7, Cxcl1, and Cxcl10. Their upregulation correlated strongly with peak photoreceptor death, at 24 hrs exposure. *In situ* hybridization revealed that the modulated chemokines were expressed by a combination of Müller cells, activated microglia, and retinal pigment epithelium (RPE). This preceded large increases in the number of CD45^+^ cells at 3- and 7-days post exposure, which expressed a corresponding repertoire of chemokine receptors.

**Conclusions:**

Our data indicate that retinal degeneration induces upregulation of a broad chemokine response whose expression is coordinated by Müller cells, microglia, and RPE. The findings inform our understanding of the processes govern the trafficking of leukocytes, which are contributors in the pathology of retinal degenerations.

## Background

Chemokines are a large family of genes whose potent chemoattractant properties help drive the recruitment of leukocytes during immune surveillance and inflammation. When induced, chemokines form gradients that establish directional cues for leukocytes - such as monocytes - to sites of injury, and aid their arrest and extravasation into the parenchyma [1]. Chemokines are grouped according to the relative position of their first N-terminal cysteine residues, comprising C (γ chemokines), CC (β chemokines), CXC (α chemokines), and CX3C (δ chemokines) families [2,3]. They exert their biological activity through binding cell surface chemokine receptors, which are part of the superfamily of seven transmembrane domain receptors consisting of C, CC, CXC, and CX3C receptor subclasses [2].

Monocyte recruitment is a well-characterized feature in retinal pathologies including age-related macular degeneration (AMD) [4-7], retinitis pigmentosa [5], retinal detachment [8,9], glaucoma [10-12], and diabetic retinopathy [10,13]. In some instances such recruitment proves more detrimental than fortuitous, and monocyte aggregation is directly implicated in retinal models of neovascular-AMD [14], light-induced damage [15-17], diabetic retinopathy [18,19], and glaucoma [20,21]. Chemokine signaling is believed to play a role in mediating monocyte migration in several CNS disorders including multiple sclerosis, Alzheimer’s disease, and brain ischemia and trauma (reviewed in [2,22-24]). In the retina, upregulation of α and β chemokines, such as Ccl2, Cxcl1, and Cxcl10 have been detected by gene expression analyses in both wet and dry forms of age-related macular degeneration (AMD) [25]. Ccl2 is particularly well-characterized in the retina, and knockout studies indicate that ablation of Ccl2 or its receptor Ccr2 reduces monocyte infiltration and retinal degeneration in experimental choroidal neovascularization (CNV) [26,27] and in light-damaged Cx3cr1^−/−^ mice [28]. Additionally, we have shown that expression of Ccl2 is upregulated in Müller cells in light-induced retinal degeneration [29], and targeted knockdown of Ccl2 with siRNA reduces recruitment of microglia/monocytes and photoreceptor death [30].

Despite a growing understanding of the Ccl2 axis in relation to retinal dystrophies, there is a relative paucity in knowledge of the greater chemokine milieu of the retina and the cellular events that contribute to their expression. In this study, we aimed to investigate transcriptional regulation and spatiotemporal distribution of the chemokine response in the retina *in situ*, following light-induced degeneration. We find that multitude α and β chemokines are upregulated following light damage, in correlation with photoreceptor death. Further, we show, using *in situ* hybridization, that a coordinated trio of Müller cells, retinal pigment epithelium (RPE), and microglia express a suite of α and β chemokines following injury, for which recruiting CD45^+^ cells bear corresponding chemokine receptors.

## Methods

### Animals and light damage paradigm

All experiments conducted were in accordance with the ARVO Statement for Use of Animals in Ophthalmic and Vision Research; the study was approved by the Animal Experimentation Ethics Committee (AEEC) of the Australian National University (R.BSB.05.10). Young adult Sprague–Dawley (SD) rats aged were exposed to 1,000 lux of light damage (LD) following a previous protocol [31]. Animals were exposed to LD in increments of 1, 3, 6, 12, 17, or 24 hrs. Additionally, some animals were returned to dim-light (5 lux) conditions immediately following 24 hrs of LD for a period of 3 or 7 days, to assess post exposure changes. All time points were compared back to age-matched, dim-reared animals.

### Tissue collection and processing of whole retinas

Animals were euthanized using an overdose of barbiturate, which was administered via intraperitoneal injection (Valabarb; Virbac, NSW, Australia). The left eye from each animal was marked for orientation then enucleated for cryosectioning (n = 6), while the retina from the right eye was excised through a corneal incision for RNA extraction (n = 6). Retinas were processed using previously described protocols [31].

### Fluorescence-activated cell sorting of retinal microglia

Rats at each time point were euthanized as described previously. Retinas from both eyes were promptly removed through a corneal incision. Retinas from each animal were pooled and immediately placed in chilled Hank’s balanced salt solution (HBSS) (n = 5 per time point) and then subjected to light mechanical separation using a razor blade. Samples were transferred into 0.2% papain digestion cocktail as described in a previous protocol [32] with minor modifications, and incubated at 8°C for 45 minutes, then 28°C for 7 minutes. The resulting homogenate was centrifuged at 250 g for 5 minutes at 4°C, and the pellet was resuspended in neutralization buffer [32]. The homogenate was centrifuged again at 420 *g* for 5 minutes at 4°C, and the pellet resuspended in staining buffer containing 1.0% bovine serum albumin (BSA), and 0.1% azide. The samples were incubated in staining buffer containing a CD45 antibody conjugated to Alexa 647 (Biolegend, San Diego, CA, USA) for 45 minutes at 4°C, then washed twice in HBBS and resuspended in staining buffer. The resultant CD45-stained samples were run through a fluorescence-activated cell sorter (FACS) (BD FACSAria II; BD Biosciences, Franklin Lakes, NJ, USA). Viability of the sorted cells was assessed by labeling with DAPI. The isolated CD45^+^ cells were collected in staining buffer and kept chilled on ice until RNA extraction could be commenced.

To prepare for RNA extraction, isolated samples were centrifuged at 420 g for 5 minutes at 4°C, and the supernatant removed. RNA extraction was performed with a combination of TRIzol Reagent (Life Technologies, Carlsbad, CA, USA) and an RNAqueous-small scale kit (Life Technologies, Carlsbad, CA, USA) utilized in tandem to extract and purify the RNA respectively, as described previously [33]. Isolated total RNA was analyzed for quantity and purity with a ND-1000 spectrophotometer (Nanodrop Technologies, Wilmington, DE, USA).

### Microarray experimentation and analysis

Microarray analysis of RNA from whole retinas was conducted using raw microarray data derived from an investigation by our group [31], using Rat Gene 1.0 ST arrays (Affymetrix, Santa Clara, CA, USA); the microarray data is accessible from the NCBI Gene Expression Omnibus repository (GSE22818). Analysis compared samples from dim-reared and 24-hrs of LD experimental groups (n = 3 for each). The microarray data was analyzed with Partek Genomics Suite 6.4 software (Partek Inc., St. Louis, MO, USA), using the same parameters as in our previous investigation [31].

Statistical analysis was conducted using the analysis of variance (ANOVA) statistic with the threshold of significance set at *P* <0.05, with a differential expression cut-off of >50%. The differentially expressed genes were screened for those pertaining to chemokine activation, and were grouped according to pathway information summarized from the Gene Ontology Consortium [34].

### Polymerase chain reaction

Quantitative real-time polymerase chain reaction (qPCR) was used to validate the expression of genes identified in the microarray analysis, over the protracted light damage time course (n = 6). First-strand cDNA synthesis was performed as described previously [29]. Expression was measured using commercially available TaqMan hydrolysis probes (Life Technologies, Carlsbad, CA, USA); the particulars are provided in Table 1. The hydrolysis probes were applied in the same fashion as our previous study [29]. Fold change was determined using the ΔΔC_q_ method, where the expression of the target gene was normalized relative to the expression of the reference gene glyceraldehyde-3-phosphate dehydrogenase (GAPDH). Expression of GAPDH does not change with respect to retinal light damage, as indicated by several investigations [31,35,36].

**Table 1:** **Taqman hydrolysis probes**

**Gene symbol**	**Gene name**	**Catalog**	**Entrez Gene ID**
CCL3	Chemokine (C-C motif) ligand 3	Rn00564660_m1	NM_013025.2
CCL4	Chemokine (C-C motif) ligand 4	Rn00587826_m1	NM_053858.1
CCL7	Chemokine (C-C motif) ligand 7	Rn01467286_m1	NM_001007612.1
CXCL1	Chemokine (C-X-C motif) ligand 1	Rn00578225_m1	NM_030845.1
CXCL10	Chemokine (C-X-C motif) ligand 10	Rn01413889_g1	NM_139089.1
CXCL11	Chemokine (C-X-C motif) ligand 11	Rn00788262_g1	NM_182952.2
ADAM17	ADAM metallopeptidase domain 17	Rn00571880_m1	NM_020306.1
IL1B	Interleukin 1β	Rn00580432_m1	NM_031512.2
MYD88	Myeloid differentiation primary response gene 88	Rn01640049_m1	NM_198130.1
TLR2	Toll-like receptor 2	Rn02133647_s1	NM_198769.2
TNF	Tumor necrosis factor	Rn00562055_m1	NM_012675.3
SIGIRR	Single immunoglobulin and toll-interleukin 1 receptor (TIR) domain	Rn01501616_g1	NM_001024887.1

Standard PCR was performed on RNA samples purified from FACS-isolated monocytes/microglia, using primers specific to chemokine receptor genes (Table 2). Primers were designed using the Primer3 web-based design program [37], and tailored to transverse an intron splice site. First-strand cDNA synthesis was performed from 50 ng of RNA using the Tetro cDNA synthesis kit (Bioline, London, UK), and applied according to the manufacturer’s instructions. Standard PCR was then conducted on the samples using MyTaq DNA polymerase; the presence of PCR product and specificity of the reaction were assessed by gel electrophoresis.

**Table 2:** **Standard PCR primers**

**Gene symbol**	**NCBI RefSeq**	**Forward primer (5′ - 3′)**	**Reverse primer (5′ - 3′)**
Ccr1	NM_020542.2	GTTGGGACCTTGAACCTTGA	TGTGGTTGTGGGGTAGGTTT
Ccr2	NM_021866.1	CCAGTGTGAAGCAAATTGGA	TGGAAAATAAGGGCCACAAG
Ccr5	NM_053960.3	GTCAAACGCTTCTGCAAACA	CTTGTTCCCAGCCTTCTCAG
Cxcr2	NM_017183.1	CAGAGACTTGGGAGCCACTC	TCAGCAAAGTCACCAGAACG
Cxcr3	NM_053415.1	AAGTTCCCAACCACAAGTGC	GGCAGGAAGGTTCTGTCAAA

### *In situ* hybridization

A number of chemokines (Ccl3, Ccl4, Ccl7, Cxcl1, and Cxcl10) were cloned from PCR products (540-bp, 212-bp, 540-bp, 487-bp, and 504-bp amplicons, respectively) using cDNA prepared from rat retinas (as described in the qPCR section). Cloning was performed using the pGEM-T DNA vector system (Promega, Madison, WI, USA) and JM109 competent *E.coli* (Promega, Madison, WI, USA). A DIG RNA Labeling Kit SP6/T7 (Roche, Basel, Switzerland) was used to transcribe linearized plasmid and generate DIG-labeled antisense and sense riboprobes. The *in situ* hybridization was performed using a previously established protocol [38]; individual riboprobes were hybridized overnight at 57°C and then washed in decreasing concentrations of saline sodium citrate (pH 7.4) at 60°C. The bound probe was visualized with either NBT/BCIP or HNNP/Fast-Red (Roche, Basel, Switzerland).

Following hybridization, sections stained with HNNP/Fast-Red were also counter-stained using immunohistochemistry. *In situ*-stained sections were incubated with primary antibody overnight at 4°C, which was raised against either IBA1 (1:500; Wako, Osaka, Japan), Vimentin (1:100; Sigma-Aldrich, St. Louis, MO, USA), or RPE65 (1:200; Abcam, Cambridge, UK). Sections were then incubated with biotinylated antibodies raised against either rabbit or mouse IgG’s (Life Technologies, Carlsbad, CA, USA) for 2 hrs at room temperature and followed by incubation in streptavidin conjugated to Alexa488 (Life Technologies, Carlsbad, CA, USA) for 1.5 hours at room temperature. Slides were then cover slipped with Aqua Poly/Mount (Polysciences, Warrington, PA, USA), and immunofluorescence was viewed using a Zeiss laser scanning microscope (Zeiss, Oberkochen, Germany), and acquired using PASCAL software (Zeiss, v4.0).

### Analysis of cell death

TUNEL labeling was used to quantify photoreceptor apoptosis in cryosections, and utilized a protocol that has been documented previously [39]. Counts were made of TUNEL positive cells in the outer nuclear layer (ONL), and were performed along the full-length of retinal sections cut in the vertical meridian (superio-inferior), including the optic disc. The final count from each animal is the average at comparable locations in two nonsequential sections.

### Statistical analysis

Statistical analysis was performed using the Kruskal-Wallis one-way analysis of variance, with Dunn’s multiple comparison post-test applied where desired; differences with a *P* value <0.05 were considered statistically significant.

## Results

### Microarray analysis for chemokine-related genes following 24 hours of light damage

Analysis of microarray data compared gene expression in retinas of animals reared in dim-light conditions with those exposed to 24 hrs of LD. The reliability of the microarray data was assessed with hierarchical clustering and principal component analysis in our previous investigation [31]; both analyses indicated high reproducibility for samples in their respective treatment conditions. From the microarray data, a list of differentially expressed chemokine-related genes (*P* <0.05) was compiled (Table 3), which were grouped according to their functional roles outlined in the gene ontology consortium [34].

**Table 3:** **Differentially expressed chemokine related genes following 24 hrs LD exposure**

**Gene title**	**Gene symbol**	**Differential expression (%)**	**Affymetrix probe set ID**
**Chemokine activity (GO:0008009)**			
**Chemokine (C-C motif) ligand 2**	**Ccl2**	3609.72	10736700
		2968.75	10736701
		3683.67	10736699
**Chemokine (C-C motif) ligand 3**	**Ccl3**	1137.80	10745678
		4996.67	10745679
		2146.75	10745680
		748.47	10745681
		306.31	10745682
**Chemokine (C-C motif) ligand 4**	**Ccl4**	328.99	10736866
		247.01	10736865
		279.34	10736864
**Chemokine (C-C motif) ligand 7**	**Ccl7**	1166.78	10736704
		752.47	10736705
**Chemokine (C-C motif) ligand 9**	**Ccl9**	−90.89	10745664
**Chemokine (C-C motif) ligand 12**	**Ccl12**	490.33	10736714
		393.97	10736715
**Chemokine (C-C motif) ligand 20**	**Ccl20**	220.82	10924783
**Chemokine (C-X-C motif) ligand 1**	**Cxcl1**	945.55	10775901
		1559.57	10775902
		555.07	10775903
**Chemokine (C-X-C motif) ligand 6**	**Cxcl6**	−163.77	10775923
**Chemokine (C-X-C motif) ligand 9**	**Cxcl9**	53.71	10771668
**Chemokine (C-X-C motif) ligand 10**	**Cxcl10**	1464.18	10771658
		3087.93	10771659
		1553.85	10771657
**Chemokine (C-X-C motif) ligand 11**	**Cxcl11**	271.26	10771652
		439.80	10771651
		268.55	10771650
**Chemokine (C-X-C motif) ligand 16**	**Cxcl16**	145.41	10744462
**Chemokine Binding (GO:0019956)**			
**Chemokine (C-C motif) receptor 5**	**Ccr5**	58.98	10914620
**Chemokine (C-X-C motif) receptor 7**	**Cxcr7**	−60.16	10925293
**Positive regulation of chemokine production (GO:0045080/GO:0032722)**			
**ADAM metallopeptidase domain 17**	**Adam17**	84.01	10889348
		54.93	10889357
		124.88	10889340
		82.72	10889346
		55.11	10889355
**Interleukin 1β**	**Il1β**	210.61	10849843
		192.00	10849844
**Myeloid differentiation primary response gene 88**	**Myd88**	152.30	10920861
		261.01	10920862
		224.07	10920863
		144.33	10920866
**Nucleotide-binding oligomerization domain containing 2**	**Nod2**	−52.42	10809667
**Toll-like receptor 2**	**Tlr2**	123.65	10823972
**Tumor necrosis factor (TNF superfamily, member 2)**	**Tnfα**	106.36	10828025
		119.80	10828023
		111.45	10828024
**Negative regulation of chemokine production (GO:0045079/GO:0032682)**			
**Single immunoglobulin and toll-interleukin 1 receptor (TIR) domain**	**Sigirr**	71.95	10726694
		92.00	10726700

Chemokine ligands (GO:0008009) were the most prominent among the differentially expressed genes and included upregulation in a multitude of α (Ccl2, Ccl3, Ccl4, Ccl7, Ccl12, Ccl20), and β (Cxcl1, Cxcl9, Cxcl10, Cxcl11, Cxcl116) chemokines. Conversely, expression of Ccl9 and Cxcl6 was found to decrease following LD. Another highly represented group comprised genes involved in promoting chemokine synthesis (GO:0045080/GO:0032722), which included upregulation in Il1β, Tnfα, Adam17, Tlr2, and Myd88, and a decrease in Nod2. There was also an increase in expression of Sigirr, which is associated with inhibition of chemokine synthesis (GO:0045079/GO:0032682). Expression of chemokine receptors (GO:0019956) showed little wide modulation, although an increase in Ccr5 was observed, and the expression of Cxcr7 was reduced somewhat.

**Figure 1:**
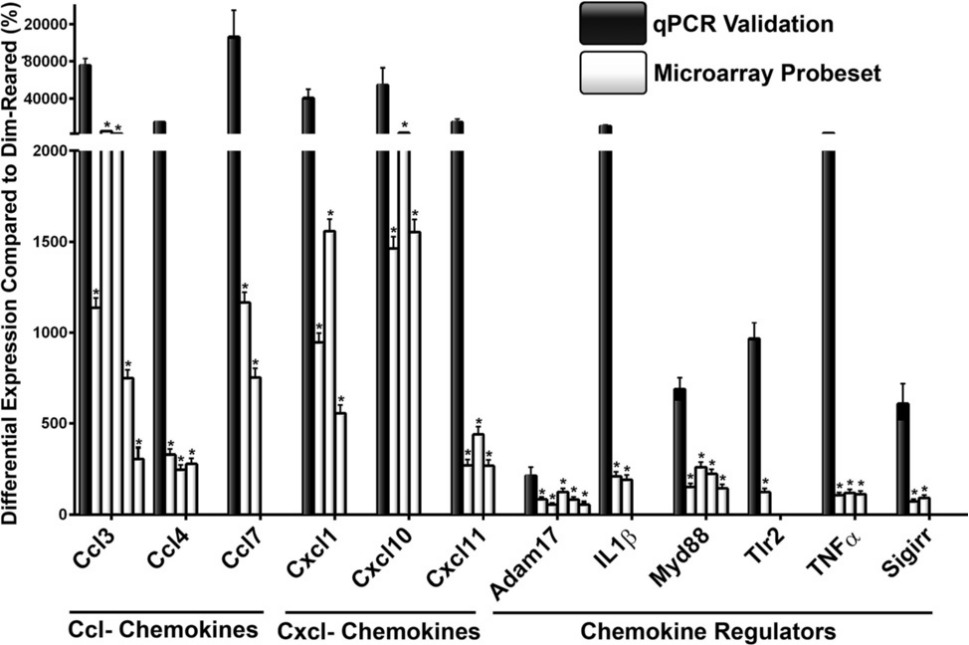
**Validation of differentially expressed microarray genes using quantitative real-time polymerase chain reaction (qPCR).** To assess robustness of the microarray data, a selection of differentially expressed microarray genes (‘*’ for each probe set, where *P* <0.05) were contrasted with their qPCR counterparts (n = 6). Increases were observed in all in genes by qPCR, which corroborated the microarray analysis, though there was some variation in the extent of upregulation.

The validity of the microarray data was assessed by analyzing the expression of 12 genes from Table 3 using qPCR. These included a selection of chemokine ligands (Ccl3, Ccl4, Ccl7, Cxcl1, Cxcl10, Cxcl11) and regulators (Il1β, Tnfα, Adam17, Tlr2, Myd88, Sigirr). Differential expression of these genes at 24 hrs of LD, was found by PCR to be in agreement with the corresponding data obtained from the microarray (Figure 1).

### Relation of chemokine expression to photoreceptor cell death

**Figure 2:**
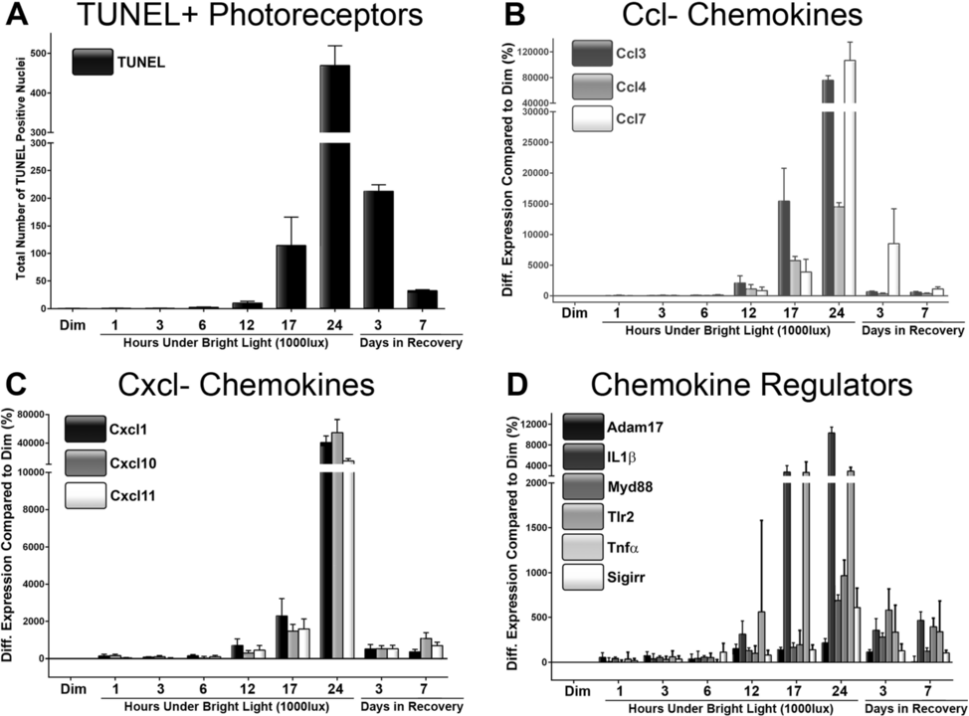
**Expression of selected chemokines and chemokine-modulators in relation to apoptotic markers, following light damage. A**: Increases in TUNEL+ photoreceptors were observed from 12 hrs light damage (LD), and peaked at 24 hrs. The number of TUNEL+ nuclei then decreased profoundly during the post exposure period. **B**-**C**: Expression of both Ccl- **(B)**, and Cxcl- **(C)** ligands exhibited concurrent upregulation beginning at 12 hrs of LD, which then peaked at 24 hrs. Expression decreased sharply in all chemokines by 3- and 7-days postexposure. **D**: Chemokine regulators Il1β, TNFα, Tlr2, Myd88, and Sigirr showed emerging upregulation between 12 hrs and 24 hrs of LD, and was particularly robust in the instance Il1β, and TNFα. Upregulation of Il1β, TNFα, Tlr2, Myd88, and Sigirr peaked at 24 hrs of LD and tapered off during the post-exposure period. Adam17, in contrast to the other regulators, exhibited consistent upregulation over the entire time course, with a minor peak at 24 hrs of LD. The trend in expression for all genes, as well as TUNEL, was significant by one-way ANOVA (*P* <0.05); n = 6.

The expression of the 12 gene selections was further examined over the protracted LD time course, and contrasted with the number of TUNEL+ photoreceptors (Figure 2). The time course encompassed incremental periods during- (1, 3, 6, 12, 17, and 24 hrs) and post-LD exposure (3 and 7 days).

Large increases in the number of TUNEL+ photoreceptors were observed from 12 hrs of LD onward and reached a peak at 24 hrs (Figure 2A), as reported previously [29,31]. By 3- and 7-days post-exposure, the number of TUNEL+ nuclei had decreased substantially. Expression of α (Ccl3, Ccl4, Ccl7), and β (Cxcl1, Cxcl10, Cxcl11) chemokine ligands was upregulated at 12 hrs of LD, before reaching a peak at 24 hrs - consistent with the emergence of TUNEL+ cells (Figure 2B-C). By 3-days postexposure, expression of all chemokine ligands was reduced, with only small upregulation evident by 7 days, compared to dim-reared controls. Expression of chemokine promoters Il1β, TNFα, Tlr2, and Myd88 and the inhibitor Sigirr exhibited similar trends in upregulation over the LD time course to the chemokine ligands (Figure 2D). Il1β and TNFβ were the most strongly upregulated, beginning at 12 hrs of LD; this upregulation decreased in the post exposure period, although the decreases were smaller for Tlr2 and Myd88. In contrast, Adam17 was upregulated over the entire time course, with a modest peak in expression at 24 hrs of LD.

### *In situ* localization of chemokines following light damage exposure

**Figure 3:**
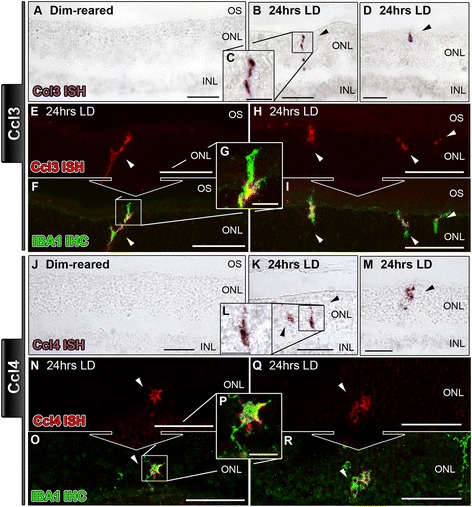
***In situ*** **hybridization for Ccl3 and Ccl4 mRNA in the retina following exposure to 24 hours of light damage.** *In situ* hybridization for Ccl3 is documented in A-I, while Ccl4 is shown in **J-R**. **A**: In retinas from dim-reared animals, staining for Ccl3 was not observed in the retina. **B**-**D**: After 24 hrs light damage (LD), there was staining for Ccl3 in irregularly shaped nuclei - ranging from elongated to globular - traversing the outer nuclear layer (ONL) of the superior retina (arrowheads). Conversely, few Cc3-expressing cells were found in the inferior retina (data not shown). **E**-**I**: Nuclei stained for Ccl3 mRNA (red) showed strong correlation (arrowheads) to activated microglia, which were counterimmunolabeled with an antibody to IBA1 (green). **J**: Ccl4 mRNA staining was not apparent in sections of dim-reared retinas. **J**-**M**: At 24 hrs, LD staining for Ccl4 was detected among clusters of irregular nuclei in the ONL from the superior retina (arrowheads); little-to-none were observed in the inferior retina (data not shown). **N**-**R**: Ccl4-expressing nuclei (red) in retinal sections show strong immunofluorescence (arrowheads) for IBA1+ microglia (green), much like Ccl3 **(E-I)**. INL, inner nuclear layer; IHC, immunohistochemistry; ISH, *in situ* hybridization; ONL, outer nuclear layer; OS, outer segments.

**Figure 4:**
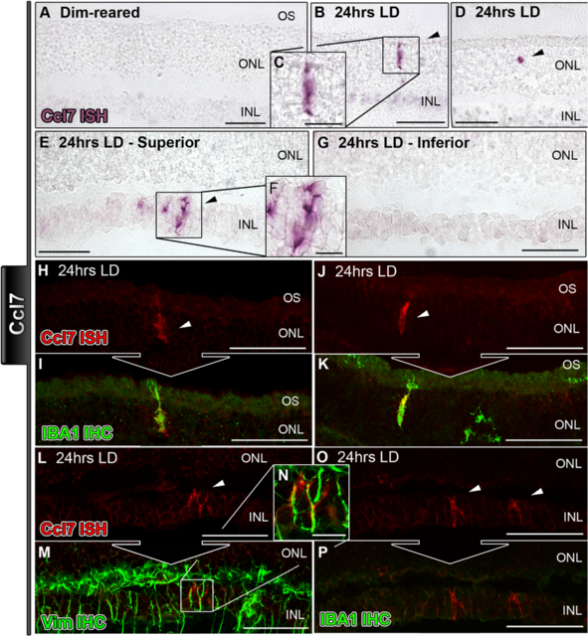
***In situ*** **hybridization for Ccl7 mRNA in the retina following exposure to 24 hours of light damage. A**: Staining for Ccl7 was not observed in the retinas of dim-reared animals **B**-**D**: In retinas exposed to 24 hrs light damage (LD), Ccl7 expression was found in infrequent clusters of nuclei within the outer nuclear layer (ONL) of the superior retina (arrowheads), with few in the inferior (data not shown). **E**-**F**: Ccl7 expression was also apparent in radial processes (arrowhead) dispersed within inner nuclear layer (INL) of the superior retina, following 24 hrs of LD. **G**: Ccl7 staining was less evident in the inferior portion of the retina following LD. **H**-**K**: Ccl7-stained nuclei (red) in the ONL were found to coincide with activated IBA1-immunolabeled microglia (green; arrowheads). **L**-**N**: Staining for Ccl7 mRNA (red) in the INL showed strong co-localization for vimentin-immunreactive Müller cell processes (arrowheads). **O**-**P**: Ccl7-expressing cells in the INL (red; arrowheads) were negative for IBA1 immunostaining. INL, inner nuclear layer; IHC, immunohistochemistry; ISH, *in situ* hybridization; ONL, outer nuclear layer; OS, outer segments.

**Figure 5:**
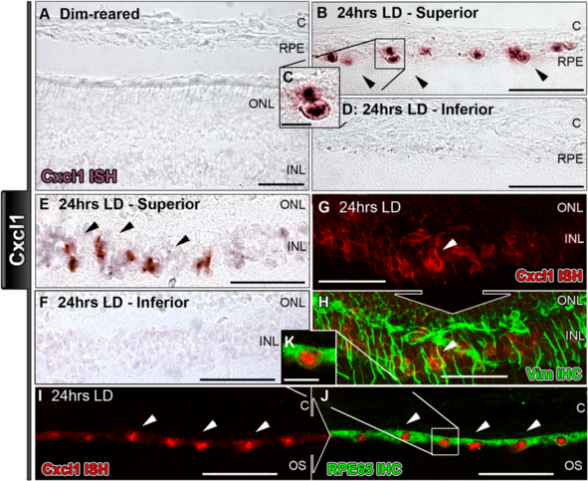
***In situ*** **hybridization for Cxcl1 mRNA in the retina following exposure to 24 hours of light damage. A**: In sections from dim-reared animals, staining for Cxcl1 was absent. **B**-**D**: Retinas exposed to 24 hrs of LD showed staining for Cxcl1 in putative retinal pigment epithelium (RPE) cells (arrowheads) in the superior retina **(B-C)**, and few-to-none in the inferior **(D)**. **E**-**F**: Staining for Cxcl1 mRNA was also present in the inner nuclear layer (INL) of superior retina following 24 hrs of LD (**E**; arrowheads), and mostly absent in inferior retina **(F)**. **G**-**H**: Retinas with stain for Cxcl1 mRNA in the INL (red; arrowhead), which was found to co-localize with vimentin-immunolabeled Müller cell processes (green; arrowheads). **I**-**J**: Localized Cxcl1 stain in putative RPE cells (red; arrowheads) correlated strongly with immunolabeling for RPE65 (green; arrowheads); Cxcl1 stain appeared to be localized in the nucleus, rather than cytoplasm. C, choroid; INL, inner nuclear layer; IHC, immunohistochemistry; ISH, *in situ* hybridization ONL, outer nuclear layer; OS, outer segments.

**Figure 6:**
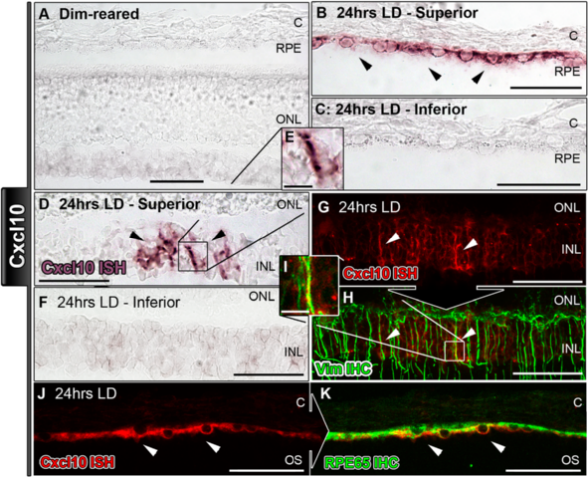
***In situ*** **hybridization for Cxcl10 mRNA in the retina following exposure to 24 hours of light damage. A**: Expression of Cxcl10 was not apparent in dim-reared sections. **B**-**C**: Sections from the superior retina **(B)** showed stain for Cxcl10 mRNA in putative retinal pigment epithelium (RPE) cells (arrowheads) following 24 hrs light damage (LD), while no staining was observed in the inferior retina **(D)**. **D**-**E**: Following 24 hrs of LD, stain for Cxcl10 mRNA appeared in the INL within superior retina (**D-E**; arrowheads). **F**: Staining for Cxcl10 was not readily apparent in the inferior retina. **G**-**I**: Sections with labeling for Cxcl10 mRNA (red; arrowheads) in the INL, which correlated with Müller cells immunolabeled with vimentin (green; arrowheads). **J**-**K**: Putative RPE cells, which were positive for Cxcl10 mRNA labeling (red; arrowheads), were also immunoreactive for the marker RPE65 (green; arrowheads). C, choroid; INL, inner nuclear layer; IHC, immunohistochemistry; ISH, *in situ* hybridization ONL, outer nuclear layer; OS, outer segments.

Chemokine ligands Ccl3, Ccl4, Ccl7, Cxcl1, and Cxcl10 were selected for further characterization in 24-hr LD retinas, by *in situ* hybridization (Figures 3, 4, 5, 6). No expression of chemokines was detected in retinas of dim-reared animals (Figures 3, 4, 5, 6). For Ccl3 and Ccl4, mRNA staining was evident by 24 hrs of LD in irregular-shaped nuclei/processes (Figure 3A-D, J-M), scattered throughout the ONL. These cells were more numerous in the superior part of the retina, which is the focal region for LD-mediated degeneration (data not shown). Counter immunolabeling for IBA1 revealed a strong co-localization of both Ccl3 (Figure 3E-I) and Ccl4 (Figure 3N-R) with activated IBA1^+^ microglia in the ONL at 24 hrs post exposure/LD.

*In situ* hybridization for Ccl7 also evident in cells scattered throughout the ONL at 24 hrs of LD (Figure 4B-D), but in addition was detected in cell processes radially oriented in the INL. As with the Ccl3 and Ccl4, this staining was more frequent in the superior retina (data not shown). Counter immunolabeling for IBA1 indicated that staining for Ccl7 in the ONL coincided with activated microglial cells (Figure 4H-K). In the INL, Ccl7 mRNA labeling of cell processes was IBA1-negative (Figure 4O-P). Instead, these cells co-localized Ccl7 mRNA with immunoreactivity for vimentin (Figure 4L-N), consistent with an identification of Müller cell processes.

The localizations of Cxcl1 and Cxcl10 resembled each other (Figures 5 and 6). Following 24 hrs of LD, Cxcl1 (Figure 5B-C) and Cxcl10 (Figure 6B-C) mRNA was labeled in putative RPE cells, predominately in the superior retina (Figures 5D, 6D). The identity of these Cxcl1/Cxcl10 expressing cells was confirmed by positive counter immunolabeling for RPE65 (Figures 5I-K, 6I-K). Cxcl1 and Cxcl10 mRNA labeling was also detected in radial processes within the INL, mainly in the superior retina, at 24 hrs of LD (Figures 5E, 6E). In this location, Cxcl1 and Cxcl10 mRNA labeling co-localized strongly with vimentin immunoreactivity consistent with an identification of Müller cell processes. (Figures 5G-H, 6G-H). A summary of these data is provided in Table 4.

**Table 4:** **Summary of retinal Ccl and Cxcl localization following light damage**

**Gene**	**Müller cells**	**IBA1+ microglia**	**Retinal pigment epithelium**
Ccl3	**-**	**+**	**-**
Ccl4	**-**	**+**	**-**
Ccl7	**+**	**+**	**-**
Cxcl1	**+**	**-**	**+**
Cxcl10	**+**	**-**	**+**

### Recruitment of CD45^+^ and expression of chemokine receptors

**Figure 7:**
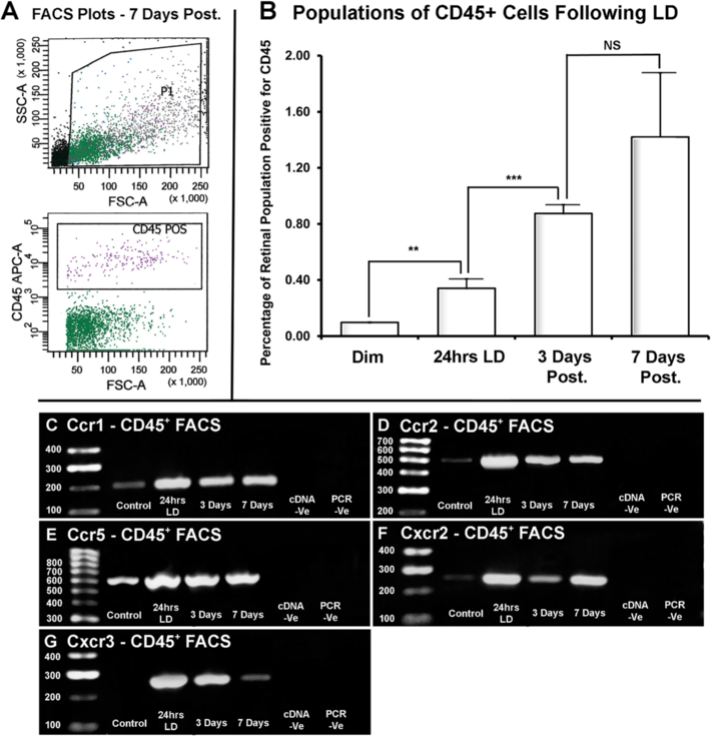
**Changes the number of CD45** ^**+**^ **cells using fluorescence activated cell sorting (FACS), and their expression of chemokine receptors. A**: Representative FACS plots, with gating strategies, for a 7-days postexposure sample stained for CD45. Gating methodology was applied equally for all samples. **B**: Histogram depicts changes in the population of retinal CD45^+^ cells following light damage (LD). Proportion of CD45^+^ cells roughly tripled following 24 hrs of LD (*P* <0.05), and continued to increase substantially during the post-exposure period after 3 days (*P* <0.05); a further increase at 7 days was not significant (*P* >0.05). The overall trend was significant by one-way ANOVA (*P* <0.05); n = 5. **C**-**G**: Representative images of PCR products via electrophoresis for Ccr1, Ccr2, Ccr5, Cxcr2, Cxcr3, in samples of CD45-sorted cells. Receptor expression was low in dim-reared control samples **(C-F)**, and absent for Cxcr3 **(G)**. Expression increased substantially following 24 hrs of LD for every receptor assessed **(C-G)**, and was maintained through the post exposure period, with the exception of Cxcr3 **(G)**.

Changes in the number of CD45^+^ monocytes/microglia following LD were identified using FACS; representative gating strategies and scatter blots are noted in Figure 7A. In dim-reared retinas, CD45^+^ cells comprised a relatively small population of the gated retinal isolates, at approximately 0.098% (Figure 7B). After 24 hrs of LD, the retinal CD45+ population rose substantially to 0.340% (*P* <0.05), then reaching 0.875% by 3 days (P < 0.05). A further increase to 1.24% was observed at 7 days, although this was not significantly different from the population size at 3 days (*P* >0.05).

PCR of RNA from the CD45-FACS isolates at each time point showed differential expression of the chemokine receptors Ccr1, Ccr2, Ccr5, Cxc2, and Cxcr3 (Figure 7), which are known to bind the differentially expressed ligands validated by qPCR in Figure 1 [40]. CD45 isolates express all chemokine receptors assessed (Figure 7C-G), although the density of the PCR bands varies depending on the time point. Ccr1, Ccr2, Ccr5, and Cxcr2 appear to be lowly expressed in dim-reared animals and more highly expressed following 24 hrs of LD and at the post-exposure time points (Figure 7C-F). Cxcr3 showed no amplification in dim-reared samples, was highly expressed at 24 hrs of LD, and was low by 7-days post-exposure (Figure 7F).

## Discussion

These findings identify a complex network of chemokine activity elicited by light-induced retinal degeneration and include several novel findings. First, we show that a suite of α (Ccl3, Ccl4, Ccl7) and β (Cxcl1, Cxcl10, Cxcl11) chemokines are upregulated following 24 hrs of LD, and these chemokines correlate strongly with the emergence of photoreceptor death and upregulation of chemokine regulatory genes (Il1β, Tnfα, Tlr2, Myd88, Adam17, Sigirr). Second, *in situ* hybridization demonstrates that Müller cells, RPE, and activated microglia comprise a ‘trio’ of cells that express select chemokine ligands (Ccl3, Ccl4, Ccl7, Cxcl1, Cxcll0) after LD. Third, we show that LD-induced upregulation of chemokines is proceeded by infiltration of CD45^+^ monocytes/microglia, which bear corresponding receptors (Ccr1, Ccr2, Ccr5, Cxcr2, Cxcr3) at 3-days postexposure.

Several investigations have previously identified upregulation in chemokine ligands following retinal light damage, including Ccl2, Ccl3, Ccl4, Ccl7, Cxcl1, and Cxcl10 through PCR and microarray analysis [35,41-43]. However, these studies did not identify the source of their expression in the retinal environment *in situ*, nor did they closely relate chemokine expression to photoreceptor cell death and regulatory factor expression. To our knowledge, this is the first investigation to demonstrate a preferential localization of chemokine mRNA in activated microglia (Ccl3, Ccl4, Ccl7), Müller cells (Ccl7, Cxcl1, Cxcl10), and RPE (Cxcl1, Cxcl10) in the degenerating retina. These observations are supported by a number of *in vitro* studies, which have reported expression of Ccl3 by microglia [43], and Cxcl1 [44-46] and Cxcl10 [47,48] in Müller cells or RPE in response to various stimuli including cytokines and bacterial pathogens*.* The expression profiles of chemokine ligands by microglia, Müller cells, and RPE following LD were highly defined and cell-specific. The precise roles of these discrete chemokine phenotypes in the degenerating retina are unclear, despite a bourgeoning understanding of chemokine activity in recent years. Functional significance may be inferred, however, based on current understanding of the chemokines identified.

### Chemokine secretion by activated microglia

We find that Ccl3, Ccl4, and Ccl7 are expressed by activated microglia after LD. Previous *in vitro* and *in vivo* studies indicate that Ccl3 is involved in the proliferation and mobilization of mature myeloid progenitor cells, as well as in recruitment of bone marrow-derived monocytes [49-52]. This function may be exerted through the binding of receptor Ccr1 [53,54], although Ccl3 also interacts with Ccr5, which mediates monocyte recruitment, and mobilization of Th1 T cells [52,55,56]. In the retina, deficiencies in Ccl3 reduce progressive photoreceptor death and monocyte recruitment in degenerative Abca4^−/−^ Rdh8^−/−^ mice, and in the Mertk^−/−^ mouse model of retinitis pigmentosa [43]. Ccl4 also interacts with Ccr5, to promote recruitment of monocytes into the retina when subjected to oxygen induced retinopathy [57].

In contrast, Ccl7 is a ligand of the better documented receptor Ccr2, and is major determinant in chemotaxis of monocytes. Deficiencies in Ccr2 impair monocyte recruitment to tissues in disease models ranging from arthrosclerosis [58] and autoimmune encephalitis [59], to choroidal neovascularization [26]. Several studies indicate that Ccl2 and Ccl7 are the primary agonists of Ccr2, in which ablation of either of these ligands reduced monocyte recruitment from bone marrow to peripheral vessels in thioglycollate-induced peritonitis [60] and *Listeria monocytogenes* infection [61]. Taken together, the findings suggest that activated ‘resident’ microglia are potent drivers of monocyte filtration from the vasculature to the parenchyma following LD. The present results suggest that this activity is mediated, at least in part, by activated microglia through the expression of specific chemokines. This expression is possibly triggered by migrating microglia that encounter stressed photoreceptors in the ONL. This suggestion is supported by our findings that recruited CD45^+^ cells bear the corresponding Ccr1, Ccr2, and Ccr5 receptors following LD and found to be expressed by monocytes and macrophages in other studies [56,62,63].

### Chemokine secretion by retinal pigment epithelium and Müller cells

In contrast to the monocyte-centric chemotactic profile of activated microglia, chemokine expression by Müller cells and RPE suggests they have a broader role in modulation of the leukocyte response. Müller cells and RPE shared a similar expression profile of upregulation of Cxcl1 and Cxcl10 following LD, while Müller cells also expressed Ccl7. We have shown previously that Müller cells also express Ccl2 following LD [29].

Cxcl1, and its cognate receptor Cxcr2, are commonly associated with recruitment of neutrophils from the vascular supplies [64-66], and signaling via this receptor is implicated in the pathogenesis of rheumatoid arthritis [67,68], lung injury [69], and adenoviral keratitis [70]. Furthermore, neutrophil recruitment contributes to pathology in choroidal neovascularization [71] and in loss of blood retinal barrier integrity in RPE-choroid explants [72]. Cxcl1 and Cxcr2 are also implicated in mobilization of monocytes, in models of atherosclerosis [73,74] and is Cxcr2 expressed by CNS- and retinal-derived monocytes [75,76].

On the other hand, Cxcl10 plays a role in chemotaxis of T cells via Cxcr3 signaling. Cxcr3 signaling is required for recruitment of cytotoxic T cells in West Nile virus encephalitis [77,78], and in the eye, Cxcl10 mediates Th1 T cell trafficking in response to chronic ocular Toxoplasmosis [79]. Besides T cells, Cxcr3 is also expressed on monocytes/microglia as described *in vitro* and *in vivo* [80-82] and may modulate their recruitment in response to noxious stimuli. Supplementation with Cxcl10 also promotes the survival of photoreceptor *in vitro* and in retinal explants, which suggest additional neurotrophic properties [48]. Neutrophil and T cell activity are poorly characterized in light-induced retinal degeneration, possibly because, in instances where it has been investigated, their numbers are relatively small compared monocytes/microglia [43]. Therefore, the precise recruitment and function of neutrophils and T cells in retinal light damage warrants further investigation.

Synthesis of Cxcl1 and Cxcl10 by RPE and Müller cells and their juxtaposition to the choriocapillaris and retinal vasculature, respectively, possibly reflects their role as efficient mediators of chemotaxis of monocytes, neutrophils, and T cells from the circulation. The additional secretion of Ccl7 by Müller cells - in conjunction with Ccl2 [29] - may suggest a stronger emphasis on monocyte recruitment from the retinal vessels following LD, consistent with observation of an influx of bone marrow-derived monocytes from the retinal vasculature in retinal injury following exposure to bright light [83].

### Factors that promote chemokine expression in the degenerating retina

We also find in this study the upregulation of factors that have broad modulatory roles in chemokine secretion, including the pro-inflammatory cytokines IL1β and TNFα. These factors induce expression of both α and β chemokines *in vitro* [84] and under various conditions including mycobacterial infection [85], nephrotoxicity [86], LPS-induced endotoxemia [87], and arthritis [88]. Other genes identified in this study that may induce chemokine expression are Tlr2 [89,90] and Myd88 [91,92].

The events leading to collective synthesis of chemokines by microglia, Müller cells and RPE following LD are uncertain, although recent evidence conducted *in vitro* points to an interactive role with microglia in chemokine secretion. Müller cells co-cultured with LPS-activated microglia were found to upregulate expression of Ccl2 and Ccl3 [93]. These microglia-stimulated Müller cells in turn induced reciprocal activation of unstimulated microglial cultures, including upregulation of Il1β. Co-culturing with activated microglia also induced upregulation chemokines in RPE cultures, such as Ccl2 and Ccl5 [94]. The data suggest that the trio of cells identified as expressing chemokines in the LD model co-activate; whether direct interaction between cell types is required is unclear, although, if so, would be most likely mediated by activated microglia, which are the only motile cell of the trio. It should be noted that *in vivo* Müller cells and RPE do not express Ccl3 and Ccl2, respectively.

## Conclusions

The study shows that microglia, Müller cells, and RPE each contribute to the trafficking of leukocytes following retinal damage through coordinated chemokine secretion. We show that chemokine expression by this trio of cells precedes mass recruitment of CD45^+^ cells, which bear the corresponding chemokine receptors and which increased in number in the retina over the time course of the experiment. Our findings provide valuable insight into chemokine activity in retinal degenerations.

## Abbreviations

Adam17: ADAM metallopeptidase domain 17
AEEC: Animal Experimentation Ethics Committee
AMD: age-related macular degeneration
C: choroid
Ccl12: Chemokine (C-C motif) ligand 12
Ccl2: Chemokine (C-C motif) ligand 2
Ccl20: Chemokine (C-C motif) ligand 20
Ccl3: Chemokine (C-C motif) ligand 3
Ccl4: Chemokine (C-C motif) ligand 4
Ccl7: Chemokine (C-C motif) ligand 7
Ccl9: Chemokine (C-C motif) ligand 9
Ccr5: Chemokine (C-C motif) receptor 5
CNS: central nervous system
Cxcl1: Chemokine (C-X-C motif) ligand 1
Cxcl10: Chemokine (C-X-C motif) ligand 10
Cxcl11: Chemokine (C-X-C motif) ligand 11
Cxcl16: Chemokine (C-X-C motif) ligand 16
Cxcl6: Chemokine (C-X-C motif) ligand 6
Cxcl9: Chemokine (C-X-C motif) ligand 9
Cxcr7: Chemokine (C-X-C motif) receptor 7
FACS: fluorescence activated cell sorting
GAPDH: glyceraldehyde-3-phosphate dehydrogenase
HBSS: Hank’s balanced salt solution
IHC: immunohistochemistry
Il1β: Interleukin 1β
INL: inner nuclear layer
ISH: *in situ* hybridization
LD: light damage
Myd88: Myeloid differentiation primary response gene 88
Nod2: Nucleotide-binding oligomerization domain containing 2
ONL: outer nuclear layer
OS: outer segments
PCR: polymerase chain reaction
qPCR: quantitative real-time polymerase chain reaction
RPE: retinal pigment epithelium
SD: Sprague–Dawley
Tlr2: Toll-like receptor 2
Tnfα: Tumor necrosis factor (TNF superfamily, member 2)
TUNEL: terminal deoxynucleotidyl transferase dUTP nick-end labeling
